# Resilient, but for how long? The relationships between temperament, burnout, and mental health in healthcare workers during the Covid-19 pandemic

**DOI:** 10.3389/fpsyt.2023.1163579

**Published:** 2023-06-29

**Authors:** Joshua Gertler, Lourdes Dale, Natasha Tracy, Joelle Dorsett, Nicola Sambuco, Andrea Guastello, Brandon Allen, Steven P. Cuffe, Carol A. Mathews

**Affiliations:** ^1^UF Center for OCD, Anxiety, and Related Disorders, University of Florida, Gainesville, Florida, United States; ^2^University of Florida, Evelyn F. and William L. McKnight Brain Institute, Gainesville, Florida, United States; ^3^Department of Clinical and Health Psychology, College of Public Health and Health Professions, University of Florida, Gainesville, Florida, United States; ^4^Department of Psychiatry, College of Medicine-Jacksonville, University of Florida, Jacksonville, Florida, United States; ^5^Department of Psychiatry, College of Medicine, University of Florida, Gainesville, Florida, United States; ^6^Department of Educational and Psychological Studies, School of Education and Human Development, University of Miami, Miami, Florida, United States; ^7^Department of Emergency Medicine, College of Medicine, University of Florida, Gainesville, Florida, United States

**Keywords:** anxiety, depression, PTSD, Covid-19 pandemic, temperament, healthcare workers, burnout, longitudinal

## Abstract

**Introduction:**

Dispositional traits of wellbeing and stress-reaction are strong predictors of mood symptoms following stressful life events, and the COVID-19 pandemic introduced many life stressors, especially for healthcare workers.

**Methods:**

We longitudinally investigated the relationships among positive and negative temperament group status (created according to wellbeing and stress-reaction personality measures), burnout (exhaustion, interpersonal disengagement), COVID concern (e.g., health, money worries), and moral injury (personal acts, others’ acts) as predictors of generalized anxiety, depression, and post-traumatic stress symptoms in 435 healthcare workers. Participants were employees in healthcare settings in North Central Florida who completed online surveys monthly for 8 months starting in October/November 2020. Multidimensional Personality Questionnaire subscale scores for stress-reaction and wellbeing were subjected to K-means cluster analyses that identified two groups of individuals, those with high stress-reaction and low wellbeing (negative temperament) and those with the opposite pattern defined as positive temperament (low stress-reaction and high wellbeing). Repeated measures ANOVAs assessed all time points and ANCOVAs assessed the biggest change at timepoint 2 while controlling for baseline symptoms.

**Results and Discussion:**

The negative temperament group reported greater mood symptoms, burnout, and COVID concern, than positive temperament participants overall, and negative participants’ scores decreased over time while positive participants’ scores increased over time. Burnout appeared to most strongly mediate this group-by-time interaction, with the burnout exhaustion scale driving anxiety and depression symptoms. PTSD symptoms were also related to COVID-19 health worry and negative temperament. Overall, results suggest that individuals with higher stress-reactions and more negative outlooks on life were at risk for anxiety, depression, and PTSD early in the COVID-19 pandemic, whereas individuals with positive temperament traits became more exhausted and thus more symptomatic over time. Targeting interventions to reduce mood symptoms in negative temperament individuals and prevent burnout/exhaustion in positive temperament individuals early in an extended crisis may be an efficient and effective approach to reduce the mental health burden on essential workers.

## 1. Introduction

Given the unique challenges and stressful events associated with the COVID-19 pandemic, healthcare workers, who by nature of their jobs were on the frontlines of the crisis, have had an increased prevalence of post-traumatic stress disorder (PTSD), anxiety, and depression compared to pre-pandemic levels (1, 2). Healthcare workers have seen demands increase while they often lack sufficient equipment and may have received minimal training on how to reduce personal risk (3). A systematic review examining international rates of mental health symptoms among healthcare workers during the pandemic found a 30.0% pooled prevalence of anxiety, 31.1% pooled prevalence of depression and depressive symptoms, and a 31.4% pooled prevalence of PTSD in this population, which are higher than population-based prevalences for these disorders [(4); Depression 24.4%, Anxiety-Including PTSD 28.2%; (5)]. While the overall strain on healthcare systems affects all healthcare workers, those treating patients with COVID-19 appear to be particularly vulnerable to worry about COVID-19, distress, and PTSD and experienced much higher levels of grief [e.g., (6, 7)]. However, as with all individuals, healthcare workers can vary in their resilience against or vulnerability for developing psychiatric symptoms (8).

The constructs of resilience and vulnerability were first developed in populations at-risk for developing PTSD, such as combat veterans, and relate to whether an individual can maintain psychological well-being in the face of stressful and life-threatening situations (9–11). Resilience also appears to be a protective factor against other internalizing disorders such as anxiety and depression in other populations (11, 12). This construct is considered an innate trait (13) with some fixed factors, including age, genetic predisposition, and marital status (14). However, external protective factors such as educational interventions, work-life balance, life experience and organizational structure can bolster resilience (15, 16). In healthcare workers during the COVID-19 pandemic, resilience has been negatively associated with levels of stress and anxiety, depression and PTSD (2, 17–23). Understanding the relationships between vulnerability/resilience to psychiatric symptomatology in the face of a global medical crisis such as the COVID-19 pandemic is therefore critical to identifying ways to mitigate downstream negative health consequences of such emergencies on healthcare personnel.

While a number of scales directly assessing skills and coping strategies related to resilience have been developed to predict risk of developing mental health disorders and treatment response [e.g., Conor-Davidson Resilience Scale: (24); Brief Resilience Scale: (25)], temperament constructs associated with resilience appear to be the strongest predictors of emotional distress (26–28). Temperament, an aspect of personality relating to individual differences in behavior style, can be described in a 3 factor model that includes *negative affect* (NA), *positive affect* (PA), and *impulsivity* (29, 30), which each show substantial heritability and long-term stability in adulthood (31, 32). Differences in these traits have been found to moderate lifetime susceptibility to mental health disorders (33), with NA and PA predicting mood and anxiety disorders (26, 34, 35), and the development of PTSD symptoms following acutely stressful events such as a hurricane (36) and long-term stressors in first responders (37, 38). In these studies, participants who scored high on PA and low on NA were less likely to develop PTSD and other mood or anxiety disorders, while those with low PA and high NA were more likely to develop symptoms. More direct measures of resilience, such as the Conor-Davidson Resilience Scale, typically correlate with PA but not NA (26). Some have proposed that vulnerability is not simply the absence of resilience, but rather the presence of other factors such as low emotional stability [i.e., trait neuroticism; (39)]. Indeed, one study of healthcare workers during the pandemic reported negative associations for psychiatric symptoms with emotional stability and resilience (40). Given the previous literature on the relevance of NA and PA to the development of psychiatric symptomatology, and the enormous strain that COVID-19 has posed on public health infrastructure and on healthcare workers (41, 42), there is a high likelihood that NA and PA are important factors in determining healthcare workers’ resilience and vulnerability against mood and anxiety disorders in the face of this pandemic.

NA and PA are also highly correlated with burnout, which is associated with anxiety and depression symptoms (42–45). High levels of stress can lead to burnout, described as a depletion of psychological resources which involves emotional exhaustion, reduced personal achievement (i.e., loss of interest in work, feelings of ineffectiveness), and depersonalization (i.e., a tendency to see people as objects rather than humans) (46, 47). Healthcare workers have historically been known to experience high rates of burnout due to their unique job stressors (48–50). In particular, work overload, long or alternate schedule working hours, lack of infrastructure support, and the emotional labor of having to hide negative emotions while on the job are just a few of the many factors contributing to burnout in healthcare workers (51–53). Unfortunately, the COVID-19 pandemic has only exacerbated rates of burnout among healthcare workers (54, 55), as many of these pre-existing stressors were only increased (e.g., increased workload, long working hours, and emotionally intense labor).

Additional stressors contributing to burnout include concerns about infecting relatives with COVID-19, confusion due to constantly changing patterns of action regarding the pandemic, the experience of seeing patients and colleagues die at increased rates and moral injury (41, 54). Moral injury occurs following a transgression against deeply held moral beliefs, such as choosing which patients receive ventilators and do not, that can produce feelings of shame, guilt, emotional distress weakened trust, reduced self-forgiveness, view of self as immoral/irredeemable in an unjust world, and suicidality (56–58). Although moral injury is separate from PTSD, it often co-occurs and contributes to symptoms of anxiety and depression among healthcare workers (59, 60). Altogether, interconnections between burnout, covid-related worry, and moral injury are likely to be key features of the distress vulnerable healthcare workers are experiencing.

This study aimed to investigate the relationships between temperament, professional burnout, moral injury, and COVID-specific factors, and symptoms of anxiety, depression, and PTSD in healthcare workers during an 8-month period near the beginning of the COVID-19 pandemic. We first used negative and positive affect measures to identify those who were more likely to be resilient to the stressors of the pandemic and thus demonstrate fewer psychiatric symptoms. We hypothesized that healthcare workers indicating low Negative and high Positive Emotionality would report lower levels of distress on measures of anxiety, depression, PTSD, burnout, COVID-related worry, and moral injury over 8 months in the early phases of the COVID-19 pandemic. We also explored the relationships among these measures to determine which were the strongest predictors of psychiatric symptoms.

## 2. Method

### 2.1. Participants and recruitment

This study was approved by the University of Florida Institutional Review Board. Details of the recruitment and methods have been previously published (41, 42) and are summarized briefly below. Participants were recruited to join the study via announcements posted throughout hospitals, clinics, nursing homes, and other medical settings in two North Central Florida cities, Gainesville and Jacksonville, and via brochures emailed to relevant departments or clinical services at the University of Florida in both cities with permission from the department head or appropriate administrator. Individuals were eligible for participation if they worked in a healthcare setting, regardless of their type of employment. Interested participants followed a link or scanned a QR code on the brochure that took them to a secure survey service, REDCap, where they provided consent to participate and subsequently recorded their responses to the survey questions. Paper versions of the survey were made available upon request. Participants who consented to participate and completed measures were compensated in an escalating manner based on the number of completed sessions, with a maximum total compensation of $220 for completion of all possible assessments over a total of 8 months. Data collection began in October 2020 and ended in August 2021, with participants completing longitudinal assessments approximately every 30 days. While it was not a predictable part of the study design, it is important to note that COVID-19 vaccines became available to high-risk medical workers in these health systems in December of 2020 and were available to all healthcare workers in January of 2020. Questions regarding vaccine status were added to the survey starting at timepoint 4.

### 2.2. Measures

#### 2.2.1. Temperament

The brief form of the Multidimensional Personality Questionnaire [MPQ-BF; (61)] was administered once in two parts, with well-being and stress reaction scales administered at time point 1, and aggression and alienation scales administered at time point 2. The MPQ-BF includes 11 primary trait scales that combine around three orthogonal higher-order factors: Positive affect, negative affect, and constraint. Previous studies have demonstrated that the well-being trait of the MPQ-BF was the most predictive of positive affect and the stress reaction trait was most predictive of negative affect (61). As these two traits represent the direct counterparts to positive and negative emotional dispositions (29, 62), only the questions referring to well-being and stress reaction were included in the current study. Twelve items composed the well-being scale, in which participants reported their agreement (true-false) to a variety of statements describing optimism, cheerful and happy dispositions, enjoying activities, and feeling good about themselves. Thirteen items composed the stress reaction trait, in which participants reported their agreement (true-false) to a variety of statements describing levels of irritability and anxiety.

#### 2.2.2. Current psychiatric symptomatology

The first eight questions of the Patient Health Questionnaire (PHQ-8) were used as a screening instrument for depression (suicidality excluded). Referring to the 2 weeks preceding the assessment, the questionnaire asks for frequency of occurrence of a series of problems, including little interest in everyday activities, sleeping troubles, appetite issues, and trouble concentrating. The score of the first eight items of the PHQ-9 ranges from 0 to 24, with a cut point of 10 suggesting high likelihood of current depression (Kroenke et al., 2009) and high internal consistency [Cronbach α = 0.88; (63)].

Symptoms of generalized anxiety (GAD) were assessed with the GAD-7, which is considered an efficient tool for screening for GAD and evaluating its severity in clinical practice and research [Cronbach α = 0.92; (64)]. Referring to the 2 weeks preceding the assessment, participants rated the frequency of occurrence of various issues, including feeling anxious or on edge, feeling annoyed or irritable, or being irritable. The total score for the seven items of the GAD-7 ranges from 0 to 21, with a cut point of 10 suggesting a high likelihood of Generalized Anxiety Disorder.

Post-traumatic symptomatology was assessed via the 8-item version of the PTSD Checklist for DSM-5 (PCL-5), a self-report measure that assesses PTSD symptoms experienced in the last month according to DSM-5 criteria [Cronbach α = 0.92; (65)]. The items assess symptoms across the four symptom clusters of; PTSD (re-experiencing, dysphoria, avoidance, and hyperarousal) on a 0 to 4 Likert scale, ranging from 0 to 32 with a cut point of 19 suggesting a high likelihood of PTSD.

#### 2.2.3. COVID-19 health worry

Participants indicated their level of COVID-19 health worry via a 4-point Likert scale (0 = *Not Worried* to 3 = *Very Worried*) summed across 7 questions about personal health and family health. A series of 4 questions about personal health asked, “How worried are you that you will: (1) Be infected while providing medical care, (2) Be infected with the COVID-19 virus in your home or community, (e.g., while at grocery store or pharmacy), (3) Become seriously ill because of COVID-19, and (4) Infect an immediate family member if you get COVID-19?” A series of 3 questions about family health ask, “How worried are you that an immediate family member: (1) Is having trouble coping with fear of getting COVID-19, (2) Will be infected with COVID-19, and (3) Will become seriously ill with COVID-19?”

#### 2.2.4. Workplace burnout

Workplace burnout was assessed via the Professional Fulfillment Index (PFI), a 16-item measure that we used to measure healthcare workers’ attitudes about their work (66). Each item is scored on a 5-point Likert scale (0 = *not at all true* to 4 = *completely true*).

The burnout scale (Cronbach α = 0.92) is made up of a work exhaustion subscale (Cronbach α = 0.90) that assesses sense of dread, physical/emotional exhaustion, and lack of enthusiasm, and the interpersonal disengagement subscale (Cronbach α = 0.90) that assesses empathy and connection with others. Given prior research found that it was important to focus on these scales separately (41), this was done in the current study.

#### 2.2.5. Moral injury

The Moral Injury Events Scale (67) assessed the level of agreement via a 6-point Likert scale (0 = strongly disagree to 5 = strongly agree) about the occurrence and anguish of moral injury (asked as two separate questions) experienced by participants themselves (self moral injury or Self MI) and observed by participants in others (others moral injury or Others MI). The perception of betrayal questions for self and other moral injury were excluded to limit the load on the participants. We focused on the internally consistent total score for Self MI (i.e., acting against moral or failing to act consistent with morals and feeling troubled by it; Cronbach α = 0.94) and Others MI (i.e., seeing something morally wrong and feeling troubled by it; Cronbach α = 0.88) when predicting burnout. For analyses focused on determining the factors related to moral injury, we grouped the participants according to whether or not they agreed that they experienced Self and Others MI.

### 2.3. Statistical analysis

#### 2.3.1. Participant demographics and attrition

Due to limited numbers of participants in each minority group, race was binarized into white non-Hispanic and other categories. The participant who identified as non-binary and the participant who had less than a high school education were excluded in demographic analyses. The exclusion of these participants in final analyses was determined by whether gender or education were significant covariates, respectively. Attrition bias for time points 1 to 2, and 2 to 8 was assessed with JMP® Pro V.16.1 for Macintosh (68). Attrition was assessed with independent samples *t*-tests for age, and chi-squared tests for binarized race, gender, and ordinal education level. All participants with complete MPQ data were included in the cluster analyses, while participants with complete MPQ data and more than one timepoint were included in the symptom trajectory analyses, and only participants with complete data at timepoints 1 (baseline) and 2, and 1 through 8, respectively, were included in the ANOVAs described below.

#### 2.3.2. Cluster analysis

Participants were clustered using MPQ well-being and stress-reaction mean scores to identify classic temperament groups typically thought of as more vulnerable and more resilient to mental health disorders. The k-means clustering method was chosen as a data-driven multivariate technique where clustering finds partitions defined by centroids, in which the sum of the squared Euclidean distance of all cases from assigned cluster centroids is minimized. Clustering was completed using Matlab R2021a for Macintosh (69). Since k-means clustering is an unsupervised technique in which the only constraint is represented by the number of pre-specified clusters (k), the optimal number of k and the corresponding classification was assessed using the Silhouette coefficient method for a number of clusters solution ranging from 2 to 10. The highest silhouette coefficient value was found for the two-cluster solution (silhouette values for different levels of k: 2 = 0.63, 3 = 0.57, 4 = 0.59, 5 = 0.57, 6 = 0.57, 7 = 0.56, 8 = 0.58, 9 = 0.55, 10 = 0.55), suggesting that this solution was the most appropriate for describing the underlying structure of the data. Mean responses on questionnaires were then compared across clusters over 8 months.

#### 2.3.3. Cluster comparisons

All cluster comparisons were conducted using JMP® Pro V.16.1 for Macintosh (JMP Pro, 2021). Normality of scales was assessed by comparing all JMP fit indices including Normal, Glog, and polynomial distributions. When the normality assumption was violated (i.e., a non-normal distribution displayed a better fit than the normal distribution, as indicated by lower AIC and BIC values), the scales were transformed. Only the moral injury subscales violated the normality assumption and, hence, log transformation was applied. Due to the biggest changes occurring between timepoints 1 and 2, and a significant reduction in power due the smaller sample of participants who completed timepoint 8, analyses of timepoints 1 through 8 and analyses timepoints 1 to 2 were both conducted. The Professional Fulfilment Index (PFI) and Moral Injury (MI) subscales were examined for the analyses using timepoints 1 to 2 but not for the analyses using timepoints 1 through 8 to reduce number of comparisons in a smaller sample at timepoint 8. To determine necessary covariates, demographic differences between clusters were assessed with chi-square tests for categorical variables and independent sample t-tests for continuous variables. Attrition differences between groups were assessed with MANOVAs for timepoints 1 through 8 and 1 to 2. For each of the self-report scales, mean scale totals were first compared between clusters in repeated measures ANOVAs. Due to non-sphericity (*X^2^* = 210 to 223, df = 20, *p < 0*.001) Greenhouse–Geisser adjusted univariate F-tests are reported for within-subject interactions when comparing all time points. The relationships between changes in implicated scales were then further examined using ANCOVAs, controlling for baseline psychiatric symptoms, age, and binarized race. Finally, to evaluate whether relationships were similar in each temperament group, we used ANCOVAs to assess the relationship between temperament, symptom change scores and change scores of other implicated scales. Follow-up pairwise t-tests were conducted for implicated scales at each time point.

## 3. Results

### 3.1. Participant demographics

Participants (*n* = 435) who completed assessments at least for the first and second timepoints (*n* = 334, 76.8%), were primarily female (82.8%), white (73.8%), highly educated (Bachelor’s or more = 87.5%) and ranged in ages from 20 to 72 skewing younger (*M* = 38.23, SD =11.56; Table 1). Participants who had complete data included 102 doctors, 94 nurses, 90 technicians, 120 non-clerical admin staff, and 29 other professions (e.g., research assistants, etc.). Dropout from baseline to 1 month follow-up significantly differed by race with a higher percentage of white participants (78.5%) completing a 1-month follow-up than non-white participants (72.03%) (*F*_[1,339]_ = 5.9, *p = 0*.016). Attrition was not significantly different for other demographic variables. Participants completing all assessments totaled 124 and were demographically similar to those responding at the 2 month timepoint (86.2% Female; 79.8% White; 87.6% Bachelor’s or more; *M* age = 38.78, SD = 11.68).

**Table 1:** Characteristics of healthcare workers completing 1 Month follow-up by temperament cluster.

	Positive Temp	Negative Temp	Group Comparison _[DF,DFerror]_
*n*	181	153	
Age	40.57 (11.99)	35.8 (10.32)	T_[1,288]_ = 3.64, *p < 0*.001
Gender (female)	81.67%	86.18%	Χ^2^_[1,330]_ = 1.58, 0.21
Education (%)			Χ^2^_[4,325]_ = 7.96, 0.09
<HS	0.6	0	
HS	7.9	9.5	
Bach	50.6	57.1	
Grad/Professional	38.8	27.2	
Other	2.3	6.1	
Race *n* (%)			
White Non-Hispanic	137 (76)	112 (73)	Χ^2^_[1,310]_ = 7.36, *p = 0*.11
Native Amer/Ala	1 (0.6)	1 (0.7)	
Asian	8 (4.4)	9 (5.9)	
Black	27 (14.9)	13 (8.5)	
Mixed	6 (2.8)	8 (5.2)	
Native Haw/Pac Isl,	1 (0.5)	0	
Other	3 (1.7)	9 (5.9)	
Hispanic	10 (5.7)	29 (19.5)	
Wellbeing (MPQ-BF)	9.36(2.13)	5.74 (2.87)	T_[1,276]_ = 12.91, *p < 0*.001
Stress-Reaction (MPQ-BF)	3.49 (2.06)	9.21 (2.10)	T_[1,320]_ = 25.02, *p < 0*.001
^*^(SD)			

### 3.2. Clusters

**Figure 1:**
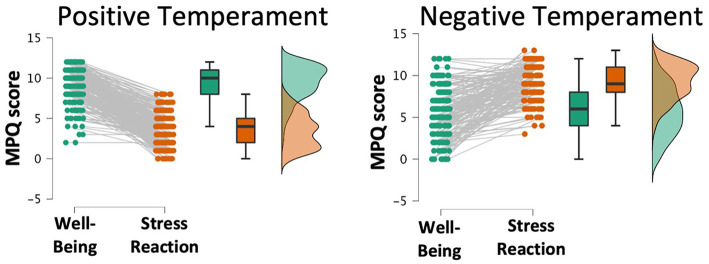
Out of 10 k-means clustering solutions on Multidimensional Personality Questionnaire subscales, a 2-cluster solution identified a Positive temperament group with high well-being and low stress reaction (left) and a Negative temperament group with low well-being and high stress reaction (right).

A k-means clustering of 2 was used to group participants based on their MPQ-BF profiles since k = 2 produced the highest silhouette value (0.63). These clusters mirrored each other. One cluster, henceforth referred to as the positive temperament group (*n* = 238), contained participants who scored higher in wellbeing (WB *M* = 9.36) than stress-reaction (SR *M* = 3.5) or were similarly low on both. The other cluster, henceforth referred to as the negative temperament group (*n* = 197), contained participants who scored higher in stress-reaction (SR *M* = 9.2) than wellbeing (WB *M* = 5.7) or were similarly high on both (Figure 1).

Dropout was not significantly different between groups at one-month follow-up (*F*_[1,432]_ = 0.14) or over all time points (*F*_[4,429]_ = 0.67), with 57% of the positive (*n* = 177) and 53% of the negative (*n* = 151) temperament groups responding at the 7-month follow-up (i.e., timepoint 8). Among those responding at 1-month follow-up, participant groups significantly differed by age (T_[1,288]_ = 3.64, *p* < 0.001) and ethnicity (Pearson *X^2^*_[1,325]_ = 14.51, *p* < 0.001), with older participants more likely to be in the positive temperament (*M* age = 40.57, SD = 11.99) than negative temperament (*M* age = 35.8, SD = 10.32) group. There was also a lower percentage of Hispanic participants in the positive temperament (*n* = 10, 5.7%) than the negative temperament (*n* = 29, 19.5%) group. Among positive temperament participants, 29 (16%) did not report their age and 3 (2%) did not report their ethnicity, while among negative temperament participants, 17 (11%) did not report their age and 4 (3%) did not report their ethnicity. Positive and negative temperament groups were demographically similar for gender (Female: positive temperament = 82%, negative temperament = 86%) and race (White: positive temperament = 76%, negative temperament = 73%).

### 3.3. Group comparisons over time

**Figure 2:**
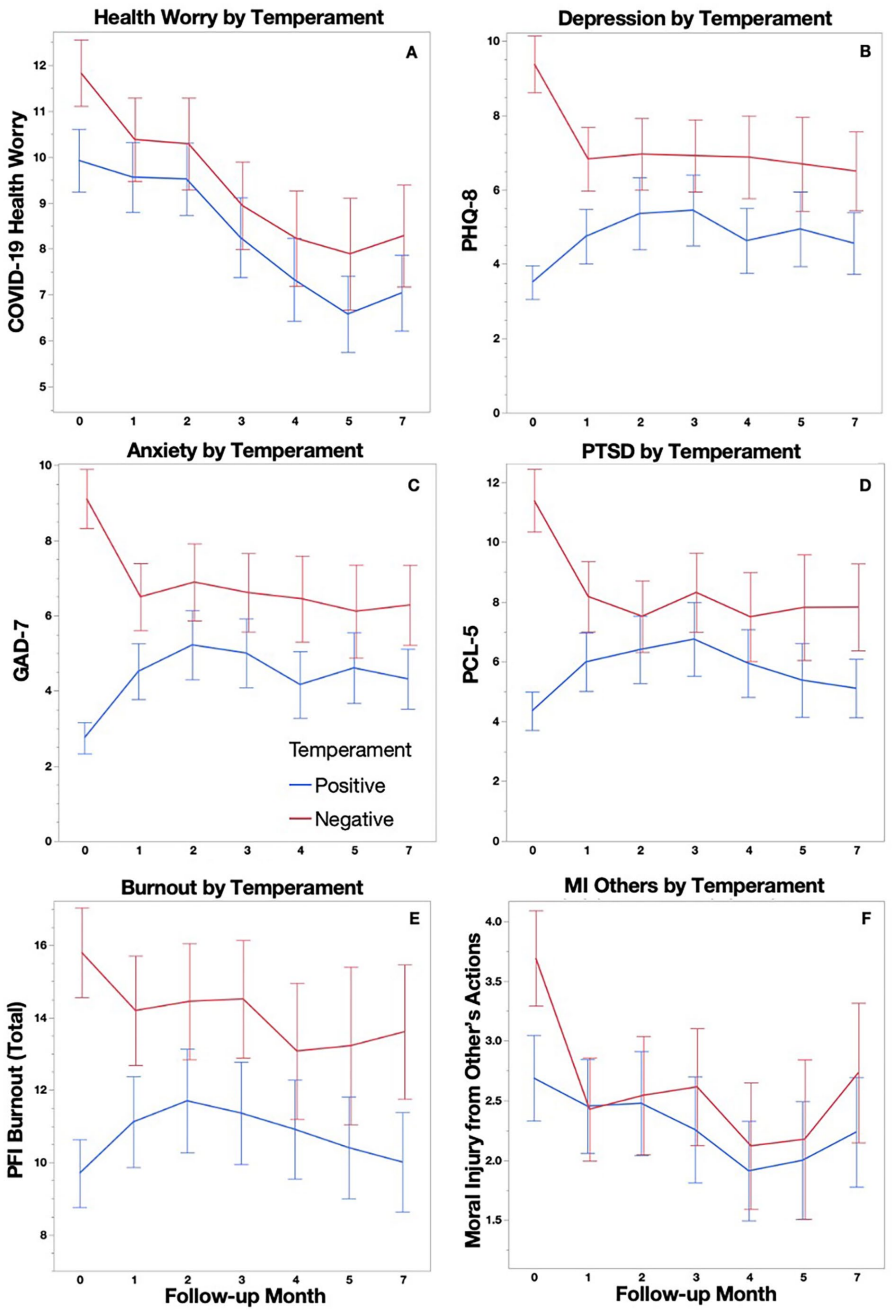
COVID-19 Health Worry **(A)**, Depression **(B)**, Anxiety **(C)**, PTSD Symptoms **(D)** Burnout **(E)**, Moral Injury from Other’s Actions **(F)** are plotted longitudinally with 95% confidence intervals for Positive and Negative temperament groups.

A repeated measures ANOVA controlling for age and binarized race/ethnicity was used to examine patterns of symptomatology and other relevant outcomes across all time points (Figure 2). Among those completing all time points, the Positive temperament group (*n* = 64) reported lower overall mean scores compared to the Negative temperament group (*n* = 60) on the PHQ-8 (*F*_[1,124]_ = 17.42, *p* < 0.001), GAD-7 (*F*_[1,124]_ = 14.63, *p* < 0.001), PCL-5 (*F*_[1,117]_ = 8.98, *p* = 0.003), Burnout scale (*F*_[1, 123]_ = 13.5, *p* = 0.001), and COVID-19 Health Worry (*F*_[1,123]_ = 3.87, *p* = 0.052), with all but COVID-19 Health Worry reaching significance after accounting for multiple comparisons (Bonferroni α_(0.05/6)_ = 0.008). COVID-19 Health Worry decreased over time for all participants (*F*_[3.26,401.4]_ = 3.06, *p* = 0.024), but this decrease was not significant after accounting for multiple comparisons (α_(0.05/6)_ = 0.008). A time by group interaction, such that scores increased for the Positive temperament group while scores decreased for the Negative temperament group, was found for the PHQ-8 (*F*_[3.38, 418.83]_ = 6.61, *p* < 0.001), GAD-7 (*F*_[3.4, 421.34]_ = 8.24, *p* < 0.001), and PCL-5 (*F*_[3.36,393.65]_ = 3.12, *p* = 0.021), with all but the PCL-5 reaching significance after accounting for multiple comparisons (Bonferroni α_(0.05/6)_ = 0.008). Moral Injury did not show between or within subject effects.

As Figure 2 indicates, the largest differences within groups occurred between baseline and 1 month follow-up, therefore, an additional analysis of these two time points alone was conducted. We were able to examine Burnout and Moral Injury subscale means separately in these analyses because increased sample sizes provided sufficient power for a greater correction of increased comparisons. The Positive temperament group (*n* = 150) reported significantly lower scores compared to the Negative temperament group (*n* = 133) at both the baseline assessment and at the 1 month follow up on the PHQ-8 (*F*_[1,274]_ = 101.06, *p* < 0.001), GAD-7 (*F*_[1,274]_ = 98.6, *p* < 0.001), PCL-5 (*F*_[1,269]_ = 69.96, *p* < 0.001), Burnout Exhaustion subscale (*F*_[1,270]_ = 37.8, *p* < 0.001), Burnout Disengagement subscale (*F*_[1,262]_ = 23.1, *p* < 0.001), and COVID-19 Health Worry (*F*_[1,279]_ = 9.29, *p = 0*.003). Age showed a negative relationship with scores on the GAD-7 (*F*_[1,274]_ = 4.96, *p* = 0.027). A time by group interaction, such that scores increased for the Positive temperament group while scores decreased for the Negative temperament group, was found for the PHQ-8 (*F*_[1,274]_ = 18.67, *p* < 0.001), GAD-7 (*F*_[1,274]_ = 29.81, *p* < 0.001), PCL-5 (*F*_[1, 269]_ = 12.40, *p* < 0.001), Burnout Exhaustion subscale (*F*_[1,270]_ = 4.34, *p* = 0.038), and the Moral Injury from Other’s Actions scale (*F*_[1,278]_ = 4.36, *p* = 0.038), with all but the Burnout Exhaustion and Moral Injury from Other’s Actions subscales reaching significance after accounting for multiple comparisons (Bonferroni α_(0.05/8)_ = 0.0063).

### 3.4. Drivers of psychiatric symptomatology at 1 month follow-up

**Figure 3:**
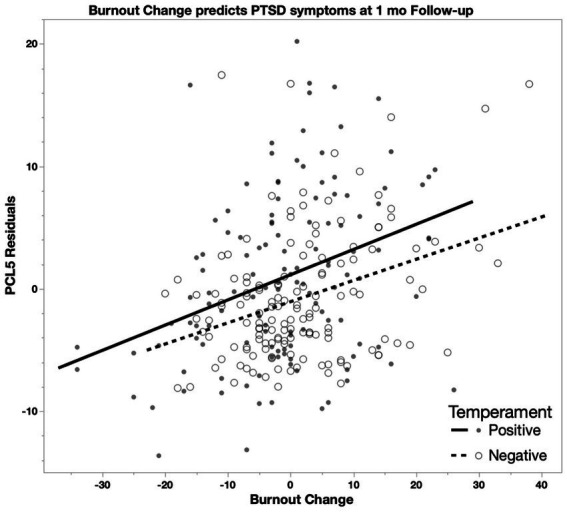
In a model predicting PCL-5 scores including changes in Burnout, COVID-19 Health Worry, and Moral Injury scores (1 month follow-up minus baseline) controlling for baseline PCL-5 score, age, and race, Burnout is positively associated with PCL-5 scores (Positive: *R^2^* = 0.12; Negative: *R^2^* = 0.11) such that higher burnout change (greater increase) predicts higher change in PCL-5 scores (greater increase).

Models predicting 1 month follow-up symptoms (PHQ-8, GAD-7, PCL-5) included temperament group, change in Burnout, change in Moral Injury and change in COVID-19 Health Worry as variables of interest (change calculated as baseline to follow-up), with baseline symptoms, age and binarized race as covariates. At 1 month follow-up, positive associations were found for the PHQ-8 with change in Burnout, GAD-7 with changes in Burnout and COVID-19 Health Worry, and PCL-5 with changes in Burnout and COVID-19 Health Worry, with all but COVID-19 Health worry predicting GAD-7 scores remaining significant after accounting for multiple comparisons (Table 2). In secondary analyses, we replaced the total Burnout and Moral Injury scores with the Burnout Exhaustion and Moral Injury by Other’s Actions subscales at a 1 month follow-up because these subscales were implicated in the repeated measures ANOVA analyses. Positive associations were found for the PHQ-8 (F_[7,264]_ = 29.56, *p* < 0.001) with Exhaustion (*F* = 77.01, *p* < 0.001), GAD-7 (*F*_[7,264]_ = 20.77, *p* < 0.001) with Exhaustion (*F* = 69.68, *p* < 0.001) and PCL-5 (*F*_[7,261]_ = 16.05, *p* < 0.001) with Exhaustion (*F* = 39.32, *p* < 0.001; Figure 3), COVID-19 Health Worry (*F* = 12.14, *p* < 0.001), and Temperament group (Positive < Negative; *F* = 6.44, *p* = 0.012) all remaining significant after accounting for multiple comparisons (Bonferroni α_(0.05/4)_ = 0.0125).

**Table 2:** Burnout, Moral Injury, and COVID-19 Health Worry Change (Time 1 to Time 2) post-hoc effects in models predicting symptoms at 1-month follow-up.

Predictors	PHQ8 (t2)		GAD7 (t2)		PCL5 (t2)	
*F*_[1,error]_	*p*	*F*_[1,error]_	*p*	*F*_[1,error]_	*p*
Burnout Δ						
Total	60.87	< 0.001	60.47	< 0.001	39.35	< 0.001
Positive Temperament	49.47	< 0.001	41.58	< 0.001	36.09	< 0.001
Negative Temperament	18.83	< 0.001	22.29	< 0.001	12.68	0.001
Covid-19 Health Worry Δ						
Total	3.22	0.074	4.59	^*^0.033	12.39	< 0.001
Positive Temperament	0.72	0.398	3.67	0.057	5.95	0.016
Negative Temperament	2.18	0.142	1.31	0.255	5.19	0.024
Moral Injury Δ						
Total	0.14	0.710	1.64	0.202	0.98	0.323
Positive Temperament	0.11	0.745	0.44	0.510	0.38	0.538
Negative Temperament	0.47	0.493	1.32	0.253	3.06	0.083

t2: 2nd time point at around a 1 month follow-up. ^*^Not significant after multiple comparison correction (Bonferroni α (0.05/4) = *p* < 0.0125).

To further examine these relationships in each group, we reran the former model (i.e., Burnout, Moral Injury, COVID-19 Health Worry) in each temperament group. At 1 month follow-up, positive associations were found for Burnout with the PHQ-8, GAD-7, and PCL-5 in both groups (Table 2), with all remaining significant after accounting for multiple comparisons (Bonferroni α_(0.05/3)_ = 0.0167). Change in COVID-19 Health Worry showed a trend level prediction of the GAD-7 in the positive temperament group and a significant prediction of the PCL-5 in both groups (Table 2). There were no significant associations for Moral Injury for either group.

### 3.5. Categorical symptom severity by temperament group

**Figure 4:**
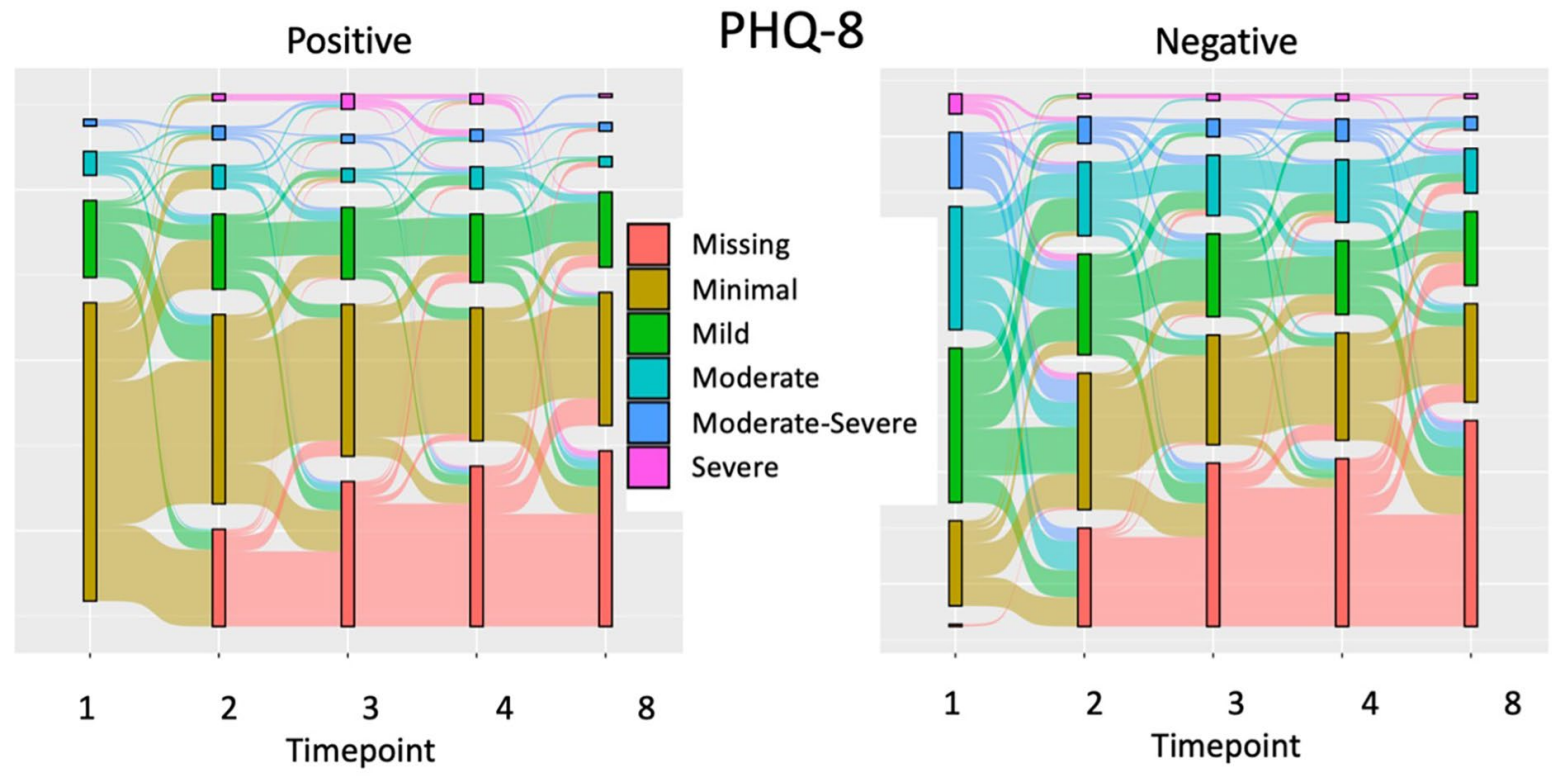
A Sankey plot of changes in Depression severity in Positive and Negative temperament groups.

While temperament findings are statistically robust, it is important to contextualize how severe symptom increases in the positive temperament group were and to what extent remission occurred within the negative temperament group. It is also helpful to contextualize symptom change relative to the maximum possible score on each scale. In the positive temperament group, the PHQ-8 increased by 4.78% (*M* = 1.15 out of 24, SD = 5.64), the GAD-7 increased by 9.19% (*M* = 1.93 out of 21, SD = 5.52), and the PCL-5 increased by 5.56% (*M* = 1.78 out of 32, SD = 7.42), while in the negative temperament group, the PHQ-8 decreased by 11.13% (*M* = 2.67 out of 24, SD = 7.23), the GAD-7 decreased by 11.57% (*M* = 2.43 out of 21, SD = 6.67), and the PCL-5 decreased by 9.38% (*M* = 3 out of 32, SD = 9.93). To display changes in severity by each participant, PHQ-8 score severity interpretations for all participants (including those missing demographic data) are plotted across timepoints 1–4, and 8 for the negative and positive temperament groups (Figure 4). All 3 symptoms scales showed similar changes in which a substantial minority (43/153) of positive temperament participants transitioned to higher clinical cutoffs from timepoints 1 to 2, and a small majority (67/132) of negative temperament participants transitioned to lower clinical cutoffs from timepoints 1 to 2.

## 4. Discussion

In a sample of healthcare workers during the COVID-19 pandemic, this study longitudinally examined differences between individuals with positive and negative temperament on scales assessing psychiatric symptoms of anxiety, depression, and PTSD, burnout, covid worry, and moral injury. Given previous studies showing that individuals with a more positive temperament are more resilient against psychiatric symptoms and other measures of distress, we predicted that this group in our sample of healthcare workers would show significantly lower scores on these scales over the 8 months studied.

As expected, the negative temperament group displayed higher (i.e., worse) scores compared to the positive temperament group across all measures at the first timepoint, however, by the third timepoint these groups were no longer significantly different. One factor that may have influenced this change is that vaccinations became available for healthcare workers in late December 2020 after the first timepoint. Indeed, as vaccinations were being rolled out in China, healthcare workers and the general population who were vaccinated reported lower levels of depression, anxiety and stress compared to those still waiting for vaccination (70, 71). In these cross-sectional studies it was unclear whether differences in pre- and post-vaccination groups was due to increased hesitancy in those with increased distress. With longitudinal data we can see significant decreases in symptoms, and while we were not able to collect vaccination information in our study, it is likely that all those who wanted to were able to get vaccinated by timepoint 3.

A decrease in symptoms for the vulnerable group was not the only change, however. Surprisingly, there was a significant interaction effect of anxiety, depression, and PTSD symptoms over the course of 8 months, particularly in the first 2 time points, driven both by an increase in symptoms for the positive and by a decrease in symptoms for the negative temperament groups. While the positive temperament group still reported significantly lower scores on psychiatric scales compared to the negative temperament group at the second time point and continued to show lower scores on average across 8 months, it is notable that a substantial number of healthcare workers in the positive temperament group transitioned from psychologically healthy to meeting generally accepted cutoff criteria for Major Depressive Disorder, Generalized Anxiety Disorder, or PTSD based on the relevant self-report measures (e.g., PHQ-8; Figure 4). It is surprising that we observed an increased risk for developing psychiatric symptoms in individuals with high wellbeing and low stress-reaction, (albeit on average still below those with low wellbeing and high stress-reaction), however, a study evaluating 18- and 19-year-olds reported a similarly surprising neuroticism and internalizing symptom interaction such that higher neuroticism was associated with smaller increases in anxiety and depression symptoms from pre-pandemic to pandemic timepoints (72). The anhedonia-apprehension scale showed a particularly stark contrast, in which high neuroticism participants not only showed no increase in severity, they scored lower on average than medium and low neuroticism individuals during the pandemic.

Despite the consistent differences between the groups characterized by positive or negative temperament with response to symptom trajectories, a large variability within these groups was also observed. In our analyses of the drivers of psychiatric symptomology at 1-month follow-up, we observed that the strongest predictor was burnout, especially exhaustion. While burnout shows a similar trajectory to psychiatric measures across groups on average, it seems to be a more robust predictor of changes in symptoms. This suggests that many individuals with a positive temperament, who were more resilient against psychiatric symptoms and burnout, could only cope with increased stressors for so long and thus experienced distress later in the pandemic than those with a more negative temperament. It is unclear what caused the increase in burnout and thus psychiatric symptoms for the positive temperament group in our study, but this finding has been reported elsewhere in Italian healthcare workers in December 2020 compared to April 2020 (73). Magnavita and colleagues reported that high workload, isolation at work, uncertainty about safety procedures, and the sharp reduction in the time devoted to meditation and relaxation contributed to the increase in their sample. A speculative explanation for the temperament interactions in our sample, based on links between temperament and life expectations (74), is that the negative temperament group expected continued stress and negative outcomes and therefore responded more positively to a modest decrease in stress following vaccination, whereas the positive temperament group viewed stressors as more temporary and therefore responded more negatively when many stressors continued post-vaccination. Alternatively, other coincident factors such as psychological and pharmacological interventions driving symptoms down in the negative temperament group may explain the interaction.

There were also additional significant predictors for PTSD symptom severity at 1 month follow-up. Change in COVID-19 Health Worry displayed an effect about 1/3 that of Burnout (based on post-hoc univariate F ratios), and temperament group displayed a small but significant effect. COVID-19 Health Worry displayed similar effects with anxiety and depression scales, but these effects did not meet rigorous statistical threshold. With many overlapping symptoms between anxiety, depression, and PTSD, it is difficult to deduce whether this pattern of results suggests a unique relationship between COVID-19 Health Worry and PTSD symptoms while GAD-7 (anxiety) and PHQ-8 (depression) scores also increase due to overlapping symptoms, or if instead, this pattern is a result of random noise such as differences in scale sensitivity or administration timing. In regard to temperament effects, only the PCL-5 was predicted by temperament group (positive < negative) suggesting that while those with a positive temperament were similarly susceptible to anxiety and depression when they reached high levels of exhaustion, they were still more resilient against PTSD. It is possible that this relates to resilience against pandemic related trauma, however, we did not assess whether events of the pandemic were the cause of this increase in PTSD symptoms. Thus, it is also possible that those with a more negative temperament were more likely to enter the pandemic with sub-clinical or mild PTSD which was then exacerbated by exhaustion, while the positive temperament group was less likely to enter the pandemic with PTSD and therefore could not display increases in PTSD symptoms other than those that overlap with anxiety and depression.

Counter to previous reports (75–77), including an analysis using data from the present study (41), moral injury did not significantly predict psychiatric symptom change in any of our models. The present analysis is unique in that we concurrently included moral injury, temperament, and burnout in the same models, and conducted analyses of variance/covariance for these variables. Future studies might investigate whether moral injury’s relationship with psychiatric outcomes is mediated by temperament and burnout. There was a trend of decreasing moral injury from others predicting decreased PTSD symptoms in the negative temperament group. Since the moral injury scale used in this study assesses both the occurrence of morally distressing events, and the impact these had on the individual, it is possible that one of these two factors does uniquely predict PTSD symptoms in vulnerable populations in addition to potential indirect effect via burnout.

The findings of this study have important clinical implications for managing the mental health of healthcare workers and other at-risk professionals during times of crisis. Those individuals with negative temperament should be targeted for intervention early on in a crisis, while those with positive temperament who initially appear resilient may need support as a crisis continues. Further, preventing or mitigating burnout, particularly exhaustion, may be the most effective way to reduce the incidence of mood and anxiety disorders in healthcare workers. One intervention recommended in 2021 was implementing “micro-practices” which combine positive psychology and mindfulness to focus on the fight-flight response, emotional exhaustion, and depersonalization (78). Other potential recommendations to consider are ensuring a psychologically and physically safe workplace, framing work as altruistic, and promoting humanism and diversity (79). Finally, clinicians might consider adapting their interventions in an extended crisis as patient characteristics change from those who are most vulnerable to those who are initially resilient but later seek treatment.

This study has important strengths, including its longitudinal design and ability to capture before and after vaccine roll-out for healthcare workers with the subsequent improvement in pandemic conditions in spring 2021. Additionally, this study included a wide sample of healthcare workers, including relatively equal numbers of assistants and technicians as well as non-clinicians, as compared to many studies investigating only doctors and nurses. This design allowed for the consideration of often over-looked yet essential members of our healthcare system, which increased the diversity of race, ethnicity, and education in our sample, improving the generalizability of our findings.

There were also some limitations to our study design. The location of our sample in the southeastern US may not generalize to other regions in the country where they may have been impacted by the COVID-19 pandemic differently. Further, these results may not generalize to unique healthcare worker positions not sufficiently sampled in this study. Another limitation is we saw a large drop in participation as time went on, and this attrition results in reduced power in analyses of later data points. Luckily, the biggest changes in scores occurred in the early time points, and we were sufficiently powered to fully explore those relationships. Finally, we were unable to examine fine-grained differences in race and ethnicity groups due limited sizes of these populations in our sample, leaving open the possibility that our results do not generalize to some of these groups.

## 5. Conclusion

Positive temperament was generally protective against internalizing symptoms in healthcare workers, at least during the time-period that began approximately 7–9 months into the pandemic in the United States. The protective effect of positive temperament dwindled over time due to both decreases in symptoms for a substantial minority with a negative temperament, and surprisingly, increases in symptoms for a majority of those with a positive temperament. This result was due to the negative temperament group beginning the study with high levels of burnout which decreased over the study period, while the positive temperament group initially displayed low levels of burnout which increased during the same period. These findings indicate that many healthcare workers with positive temperament were only protected against internalizing symptoms for so long, and developed symptoms under the extended period of stress experienced in the COVID-19 pandemic. Future studies should examine the relationship between temperament and psychiatric symptoms over longer periods of time, especially in the context of events with long lasting effects on daily life. Notably, temperament group was still protective against PTSD symptoms even with rising burnout levels. Future studies should also account for baseline PTSD symptoms by collecting information about past and recent events.

## Data availability statement

The raw data supporting the conclusions of this article will be made available by the authors, without undue reservation.

## Ethics statement

The studies involving human participants were reviewed and approved by Institutional Review Board of University of Florida (IRB Project #: 202001723; approved 15 September 2020). The patients/participants provided their written informed consent to participate in this study.

## Author contributions

NS and CM: conceptualization. JG, LD, SC, NS, AG, and CM: methodology. JG and NS: formal analysis. JG, NS, NT, JD, and LD: writing—original draft preparation. LD, CM, and BA: writing—review and editing. JG and NS: visualization. SC and CM: supervision. CM: project administration. NS, CM, and AG: funding acquisition. All authors contributed to the article and approved the submitted version.

## Funding

This research was funded by the University of Florida Clinical and Translational Science Institute, which is supported in part by the NIH National Center for Advancing Translational Sciences under award number UL1TR001427. The content is solely the responsibility of the authors and does not necessarily represent the official views of the National Institutes of Health.

## Conflict of interest

The authors declare that the research was conducted in the absence of any commercial or financial relationships that could be construed as a potential conflict of interest.

## Publisher’s note

All claims expressed in this article are solely those of the authors and do not necessarily represent those of their affiliated organizations, or those of the publisher, the editors and the reviewers. Any product that may be evaluated in this article, or claim that may be made by its manufacturer, is not guaranteed or endorsed by the publisher.
